# Upregulation of TTK expression is associated with poor prognosis and immune infiltration in endometrial cancer patients

**DOI:** 10.1186/s12935-023-03192-2

**Published:** 2024-01-09

**Authors:** Hongxiang Du, Li Zhang, Jia Chen, Xinyi Chen, Ronghui Qiang, Xiaoyi Ding, Yilang Wang, Xiaoqing Yang

**Affiliations:** 1 Department of Obstetrics and Gynecology, Affiliated Hospital of Nantong University, Medical School of Nantong University, Nantong, China; 2 Department of Cancer Research Center, Nantong Tumor Hospital, The Affiliated Tumor Hospital of Nantong University, Nantong, China; 3 Department of Oncology, The Affiliated Maternity and Child Health Care Hospital of Nantong University, Nantong, China; 4 Department of Gynaecology and Obstetrics, JiangSu XiangShui Hospital of Chinese Medicine, XiangShui, China

**Keywords:** Endometrial cancer, Threonine and tyrosine kinase, TTK, Prognosis, Immune infiltrate

## Abstract

**Background:**

Threonine and tyrosine kinase (TTK) is associated with invasion and metastasis in various tumors. However, the prognostic importance of TTK and its correlation with immune infiltration in endometrial cancer (EC) remain unclear.

**Methods:**

The expression profile of TTK was analyzed using data from The Cancer Genome Atlas (TCGA) and the Clinical Proteome Cancer Analysis Consortium (CPTAC). TTK protein and mRNA levels were verified in EC cell lines. Receiver operating characteristic (ROC) curve analysis was used to evaluate the ability of TTK to distinguish between normal and EC tissues. K-M survival analysis was also conducted to evaluate the impact of TTK on survival outcomes. Protein‒protein interaction (PPI) networks associated with TTK were explored using the STRING database. Functional enrichment analysis was performed to elucidate the biological functions of TTK. TTK mRNA expression and immune infiltration correlations were examined using the Tumor Immune Estimation Resource (TIMER) and the Tumor-Immune System Interaction Database (TISIDB).

**Results:**

TTK expression was significantly greater in EC tissues than in adjacent normal tissues. Higher TTK mRNA expression was associated with tumor metastasis and advanced TNM stage. The protein and mRNA expression of TTK was significantly greater in tumor cell lines than in normal endometrial cell lines. ROC curve analysis revealed high accuracy (94.862%), sensitivity (95.652%), and specificity (94.894%) of TTK in differentiating EC from normal tissues. K-M survival analysis demonstrated that patients with high TTK expression had worse overall survival (OS) and disease-free survival (DFS) rates. Correlation analysis revealed that TTK mRNA expression was correlated with B cells and neutrophils.

**Conclusion:**

TTK upregulation is significantly associated with poor survival outcomes and immune infiltration in patients with EC. TTK can serve as a potential biomarker for poor prognosis and a promising immunotherapy target in EC. Further investigation of the role of TTK in EC may provide valuable insights for therapeutic interventions and personalized treatment strategies.

## Introduction

Endometrial cancer (EC) is a common malignant tumor worldwide, and its incidence and mortality rates are increasing [1, 2]. Over the preceding two decades, a conspicuous increase in the incidence of endometrial cancer has been observed, with a notable predilection for impacting younger people. This form of cancer accounts for approximately 20–30% of gynecological malignancies, highlighting a worrisome trend [3]. The 5-year overall survival (OS) of patients with EC is contingent upon the diagnostic stage. Early detection often leads to favorable prognoses, with a five-year OS rate exceeding 90% [4]. Regrettably, distant metastasis is frequently encountered and significantly impacts survival rates. Once regional spread or metastasis occurs, the 5-year survival drops to 60% for stage III patients and 20% for stage IV patients [5]. Accurate and efficient evaluation of novel clinically applicable biomarkers is crucial for improving EC treatment.

Treatment strategies for endometrial cancer encompass various options, including surgery, radiotherapy, chemotherapy, and immunotherapy. Currently, the standard first-line chemotherapy for treating EC mainly involves combination therapy comprising carboplatin and paclitaxel, but half of the patients still experience chemotherapy resistance [6]. Therefore, new approaches need to be developed to enhance the sensitivity of patients to chemotherapeutic drugs in the face of drug resistance. With the integration of immuno-oncology with other immunotherapies, chemotherapies, or targeted therapies, there has been a significant transformation in treatment approaches for late-stage patients. Recently, immunotherapeutic advancements have been seen in the realm of endometrial cancer treatment, demonstrating potential therapeutic effects in clinical trials with PD-1 and PD-L1 inhibitors. Such comprehensive treatment strategies provide individual patients with more precise and effective therapeutic options [7].

Threonine and tyrosine kinase (TTK), alternatively referred to as Mps1, is a dual-specificity kinase that can catalyze the phosphorylation of both serine/threonine and tyrosine residues [8]. TTK plays a key role in safeguarding chromosome segregation fidelity, participating in the assembly of mitotic checkpoint complexes, regulating cytokinesis, responding to DNA damage, and promoting accurate chromosome alignment [9]. The spindle assembly checkpoint (SAC) represents the primary mechanism for preserving chromosomal stability during cytokinesis [10]. The SAC pathway, involving TTK, Polo, Aurora, Bub, BubR and Mad, detects misoriented chromosomes, establishes proper bipolar connections with the spindle, and diminishes chromosomal mismatch errors prior to late-stage initiation [8]. The mitotic spindle checkpoint is controlled by TTK, which monitors the tension and bipolar connections of all chromosomes attached to spindle microtubules [11]. This ensures faithful segregation of sister chromatids at the kinetochore. The cell cycle pauses during metaphase when improperly attached chromosomes are detected until all chromosomes achieve proper attachment before proceeding to anaphase [12]. In addition to regulating the mitotic SAC, TTK is involved in centrosome replication, the DNA damage response and organ development [13]. Notably, TTK is rarely detected in normal tissues except for the testis and placenta. However, TTK is overexpressed in many cancers, promoting tumor growth in glioblastoma, breast cancer and other cancers [14–16]. In triple-negative breast cancer, increased TTK expression correlates with tumor tropism and metastatic behavior [17]. Similarly, TTK knockdown in hepatocellular carcinoma inhibited tumor migration, proliferation and cellular activity. Recent evidence also indicates that TTK downregulation impedes cancer cell migration [18]. In summary, TTK has emerged as a prospective biomarker and therapeutic target for cancer.

The exact prognostic importance of TTK in endometrial cancer, as well as its connection to immune infiltration, remains incompletely understood. Given the well-documented overexpression of TTK in cancer and its ability to inhibit tumor cell migration upon TTK depletion, we developed a hypothesis suggesting a plausible correlation between TTK expression and survival outcomes in patients with endometrial cancer. To investigate and evaluate our hypothesis, we conducted a comprehensive assessment of the prognostic importance of TTK in endometrial cancer using The Cancer Genome Atlas (TCGA) dataset. In our study, we detected upregulated TTK expression in endometrial cancer. Notably, there were associations between TTK upregulation and unfavorable clinical characteristics and risk factors. Additionally, we explored the diagnostic and prognostic value of TTK in endometrial cancer as well as its correlation with immune infiltration. Our results demonstrated an association between high TTK and worse survival in patients with endometrial cancer.

## Materials and methods

### TCGA datasets

The transcriptional expression data of TTK along with pertinent clinical information were obtained from the official website of TCGA [19]. After the initial provision of RNA-Seq gene expression data in the FPKM workflow format, the data were further converted into the TPM format and subjected to log2 transformation for subsequent analysis. Given that all the data utilized in this study were obtained from the TCGA, ethics committee approval was not needed.

### RNA-sequencing data of TTK in endometrial carcinoma

The RNA-seq expression data for TTK in endometrial carcinoma were also obtained from the TCGA. A total of 545 endometrial carcinoma samples and 23 adjacent normal tissue samples were retained for analysis. The selected samples included TTK gene expression data and relevant clinical information, including age, sex, smoking status, TNM stage, and tumor location. The mRNA expression data were characterized using the mean ± standard deviation (SD) format.

### Clinical proteomic tumor analysis consortium (CPTAC), GEPIA2 and UALCAN

The CPTAC employs mass spectrometry for the analysis of tumor specimens, enabling the quantification and identification of proteins to characterize the proteome [20]. GEPIA2 uses a standardized pipeline to analyse RNA-seq data from 9,736 tumours and 8,587 normal samples in TCGA and Genotype-Ex (GTEx) cohorts [21]. UALCAN is an online tool for analysing public cancer data. We used UALCAN to evaluate TTK protein expression using CPTAC data.

### Quantitative real-time PCR (qRT‒PCR)

Total RNA was extracted from tissues, cultured cells were purified using TRIzol reagent (Invitrogen, USA), and the RNA concentration was quantified. Equal amounts of RNA (2 µg) were reverse transcribed into complementary DNA (cDNA) using Superscript Reverse Transcriptase (Applied Biosystems, USA). Quantitative real-time PCR (qRT–PCR) was performed on an ABI 7500 real-time PCR system (Applied Biosystems, USA) using PowerUP SYBR Green Master Mix (Applied Biosystems, USA). The primer sequences used for TTK and ACTB were as follows: TTK forward, 5′-TGCCCTGCGGAATTTAAACC-3′ and reverse, 5′-CTGTAAATGCCCAAGTGAACCG-3′; ACTB forward, 5′-GGCACCCAGCACAATGAAG-3′ and reverse, 5′-CCGATCCACACGGAGTACTTG-3′.

### Western blot analysis

The protein concentration was measured using a BCA kit (Pierce, USA) after lysing tissues or cells with cell lysis buffer (Cell Signaling Technology, USA) containing protease inhibitors (Roche, Switzerland). Equal amounts of protein (20 µg) were separated via 10% SDS‒PAGE and transferred to nitrocellulose membranes (Invitrogen, USA). The membranes were blocked using 5% nonfat milk at room temperature for 2 h, followed by an overnight incubation with primary antibodies at 4 °C. Following TBST washes, the membranes were incubated with HRP-conjugated secondary antibodies for 2 h at room temperature. GAPDH was used as an internal loading control. For signal detection, an enhanced chemiluminescence (ECL) kit was used, while band intensity was quantified using ImageJ software (NIH, Bethesda, USA). The primary antibodies used in this study were as follows: TTK (Santa Cruz [SANTA CRUZ], sc-56,968) and GAPDH (Proteintech, 60004-1-Ig).

### Protein–protein interaction (PPI) networks and functional enrichment analysis

STRING is a web-based database (version 11.05) that facilitates the retrieval of interacting genes and networks [22]. In our study, we utilized STRING to search for coexpressed genes and construct PPI networks, with interactions > 0.4. Gene Ontology (GO) enrichment and Kyoto Encyclopedia of Genes and Genomes (KEGG) pathway analyses provided functional insights into the coexpressed genes. The “ClusterProfiler” package was used for these analyses, and the results were visualized using the “ggplot2” package.

### Tumor immune estimation resource (TIMER) database

TIMER, an invaluable online resource, allows for the systematic analysis of immune infiltrates across various types of cancer [23]. In our study, we used TIMER to investigate the relationship between TTK expression and infiltration of immune cells, including B cells, CD4 + T cells, CD8 + T cells, neutrophils, macrophages, and dendritic cells, in uterine corpus endometrial carcinoma.

### Tumor-immune system interaction database (TISIDB)

TISIDB serves as an online web repository portal dedicated to the exploration of tumor-immune system interactions [24]. In our study, we utilized TISIDB to assess the expression of TTK and tumor-infiltrating lymphocytes (TILs) across various cancers. By leveraging the gene expression profiles, we employed gene set variation analysis to estimate the relative abundance of TILs. Spearman’s test was subsequently used to evaluate the correlation between TTK expression and the presence of TILs.

### Statistical analysis

All the statistical analyses were conducted using R (version 3.6.3). The ggplot2 package was used to visualize differences in expression. To determine the disparities between uterine corpus endometrial carcinoma tissues and adjacent normal tissues, paired t tests and Mann‒Whitney U tests were utilized. The pROC package was used to perform receiver operating characteristic (ROC) curve analysis, which identified the cutoff value for TTK [25]. K-M analysis and log-rank tests were carried out using the survminer package to evaluate the impact of TTK on survival outcomes.

## Results

### Expression of TTK from a pancancer perspective

**Fig. 1:**
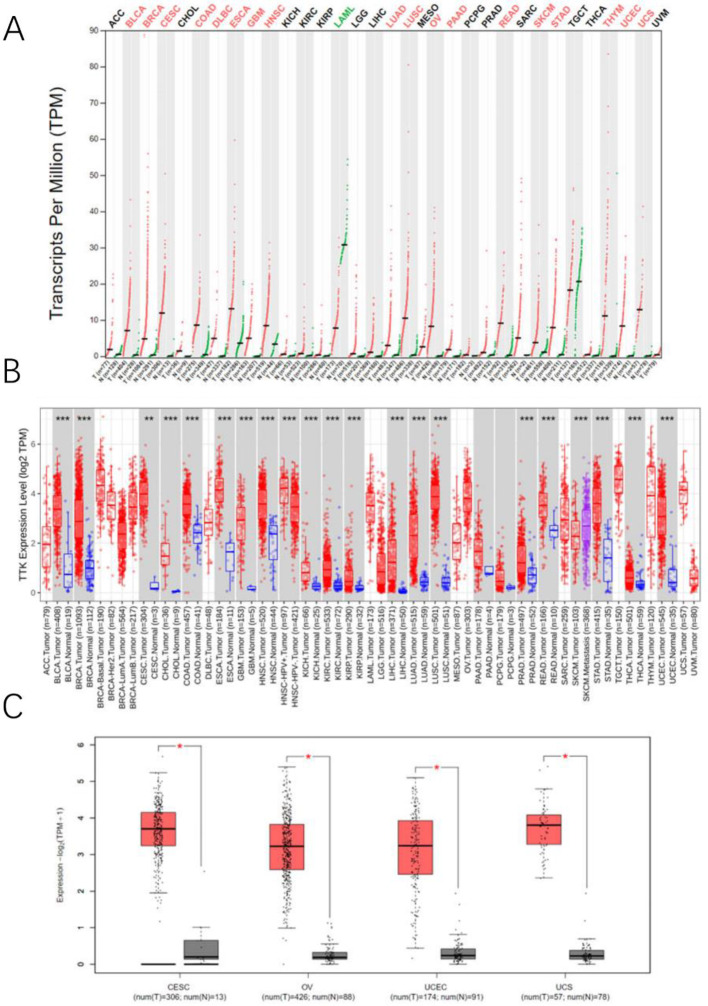
TTK expression across cancers. **(A)** TTK mRNA is upregulated in 18/33 cancer types versus normal tissues; **(B)** TIMER analysis showing upregulated TTK mRNA in 21/33 cancer; **(C)** TTK mRNA expression across cancers in the GEPIA cohort; ns = not significant; ACC, adrenocortical carcinoma; BLCA, bladder cancer; BRCA, breast cancer; CESC, cervical cancer; CHOL, cholangiocarcinoma; COAD, colon cancer; ESCA, oesophageal cancer; GBM, glioblastoma; HNSC, head and neck cancer; KICH, kidney chromophobe; KIRC, kidney renal clear cell carcinoma; KIRP, kidney renal papillary cell carcinoma; LIHC, liver cancer; LUAD, lung adenocarcinoma; LUSC, lung squamous cell carcinoma; PRAD, prostate cancer; READ, rectum cancer; STAD, stomach cancer; THCA, thyroid cancer; UCEC, uterine corpus endometrial carcinoma; UCS, uterine carcinosarcoma; UVM, uveal melanoma; ***P < 0.001

We analyzed the TTK mRNA expression profile across various cancer types. in the GEPIA database. As shown in Fig. 1A, TTK was significantly upregulated in 18/33 cancer types versus normal tissues. Similarly, the TIMER online database (Fig. 1B) showed TTK upregulation in 21/33 cancers. Taken together, these findings indicate aberrant TTK mRNA expression across many cancer types. Notably, TTK is highly expressed in gynecological cancers, including CESC, OV, UCEC and UCS (Fig. 1C).

### Upregulated mRNA and protein expression of TTK in patients and cell lines with endometrial cancer

**Fig. 2:**
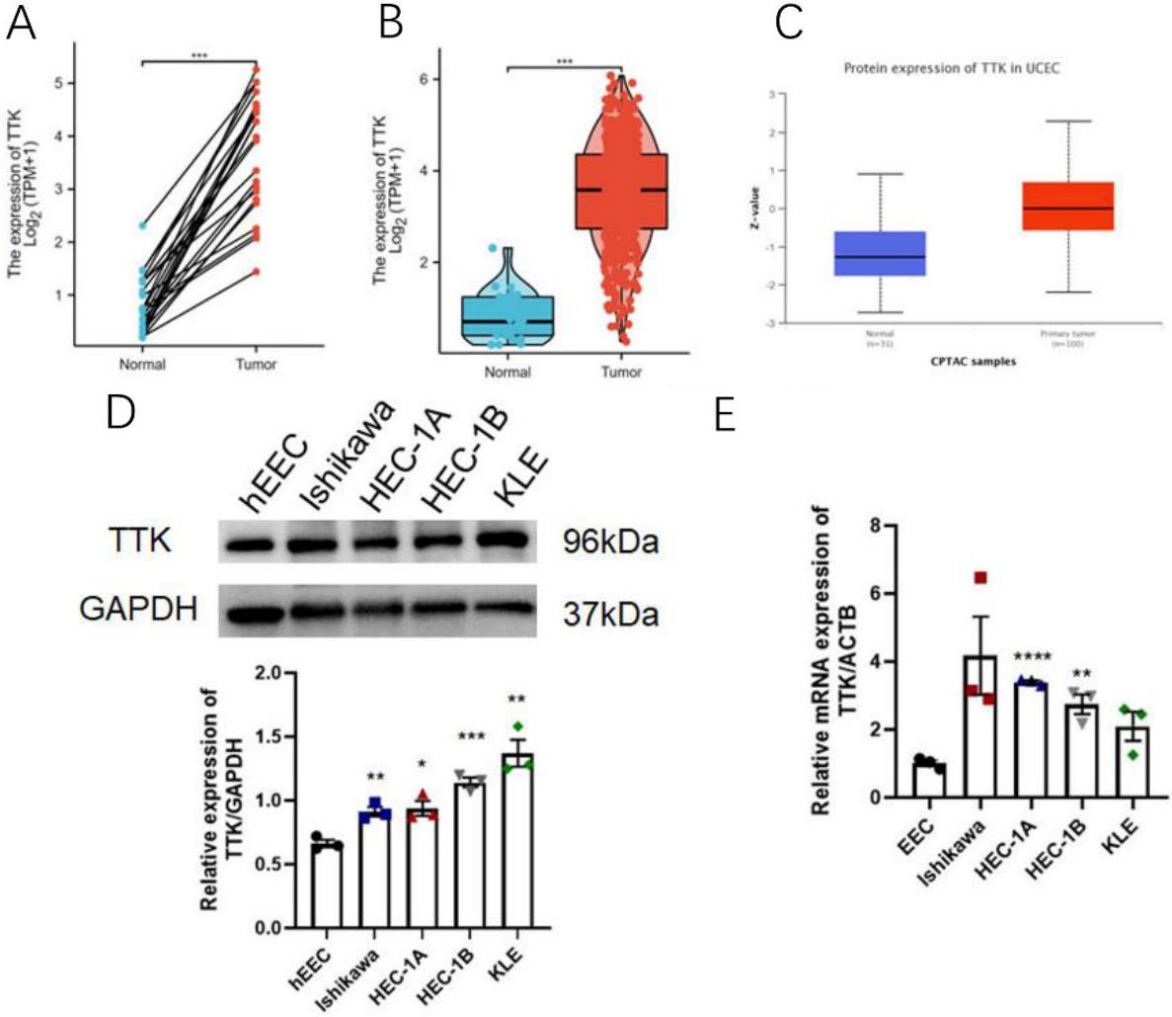
TTK expression is elevated in endometrial cancer. **(A)** paired analysis showing increased TTK mRNA in 23 tumor tissues versus adjacent normal tissues; **(B)** unpaired analysis confirmed that the TTK mRNA level was greater in 545 tumor tissues than in 23 normal tissues; **(C)** CPTAC analysis revealed increased TTK protein expression in endometrial carcinoma; **(D)** Western blot showing upregulated TTK protein in EC cell lines versus normal cells; **(E)** qPCR demonstrated elevated TTK mRNA in EC cell lines ***P < 0.001

For our study, we selected endometrial cancer as our focus. Figure 2A shows the paired analysis results demonstrating significantly increased TTK mRNA expression in 23 endometrial cancer tissues versus 23 adjacent normal tissues (p < 0.001). Likewise, Fig. 2B shows that unpaired analysis indicated significantly greater TTK mRNA expression in 545 endometrial cancer tissues than in 23 normal tissues (p < 0.001). For a comprehensive analysis of TTK protein expression, CPTAC data were utilized, and an analysis was performed using UALCAN. As shown in Fig. 2C, TTK protein expression was significantly greater in endometrial carcinoma tissues than in normal tissues. To investigate the role of TTK in endometrial cancer, we examined TTK protein and mRNA levels in endometrial cancer cell lines. Figure 2D illustrates the significant upregulation of TTK protein expression in endometrial cancer cell lines (Ishikawa, HEC-1 A, HEC-1B, and KLE) compared with that in the normal endometrial cell line (hEEC). Similarly, Fig. 2E demonstrates the significant upregulation of TTK mRNA expression in EC cell lines (HEC-1 A and HEC-1B).

### Relationship between TTK mRNA levels and clinicopathological features in endometrial cancer patients

**Fig. 3:**
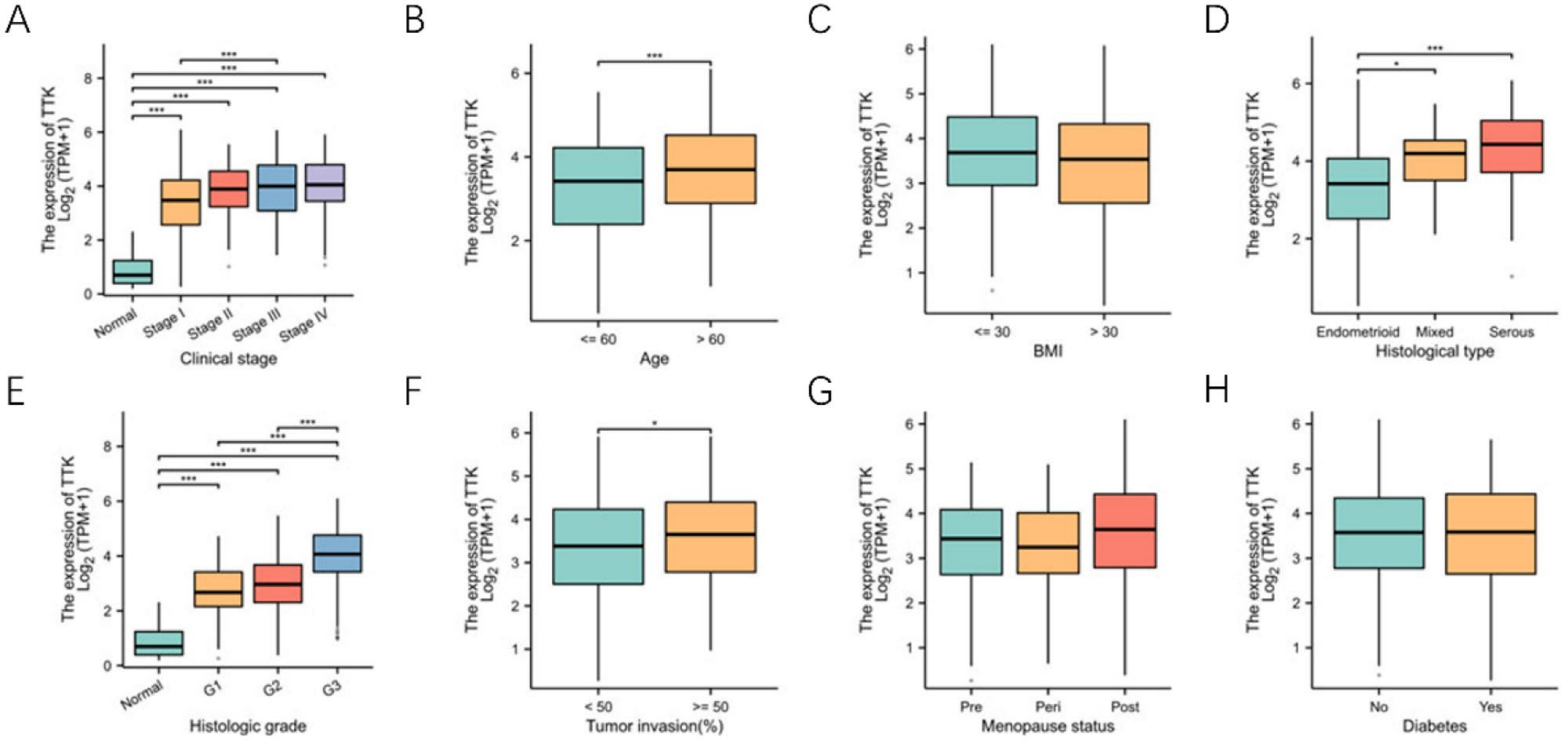
Relationship between TTK mRNA levels and clinicopathological characteristics. TTK mRNA expression was significantly correlated with the following characteristics: clinical stage **(A)**, age **(B)**, histological type **(D)**, histological grade **(E)**, and percentage of tumor infiltration **(G)**; however, the expression level of TTK was not significantly correlated with BMI **(C)**, menopausal status **(G)** or diabetes status **(H)** (*P < 0.05, **P < 0.01, ***P < 0.001)

TTK expression in endometrial cancer was examined by analyzing TTK expression data from the TCGA. The baseline characteristics of the TCGA endometrial cancer patients are presented in Table 1. The relationship between TTK mRNA expression and the clinicopathological characteristics of endometrial cancer patients was assessed using the Mann‒Whitney U test and logistic regression analysis. Table 1; Fig. 3A-H present the findings indicating increased TTK expression in patients with high clinical stage (P < 0.001), age > 60 years (P = 0.010), high histological stage (P < 0.001), plasmacytic endometrial tissue (P < 0.001), or tumor invasion ≥ 50% (P = 0.027). However, no statistically significant correlation was found between TTK expression and other clinicopathological features, such as BMI (P = 0.108), menopausal status (P = 0.209) or diabetes status (P = 0.917). The associations between TTK and tumor metastasis and high TNM stage suggest that TTK may be a poor prognostic biomarker in endometrial cancer.

**Table 1:** Clinical characteristics of the endometrial cancer patients (TCGA)

Characteristics	Low expression of TTK	High expression of TTK	P value
n	277	277	
**Clinical stage, n (%)**			< 0.001
Stage I	194 (35%)	149 (26.9%)	
Stage II	24 (4.3%)	28 (5.1%)	
Stage III	49 (8.8%)	81 (14.6%)	
Stage IV	10 (1.8%)	19 (3.4%)	
**Age, n (%)**			0.010
<= 60	118 (21.4%)	89 (16.2%)	
> 60	157 (28.5%)	187 (33.9%)	
**BMI, n (%)**			0.108
<= 30	98 (18.8%)	114 (21.9%)	
> 30	165 (31.7%)	144 (27.6%)	
**Histological type, n (%)**			< 0.001
Endometrioid	243 (43.9%)	169 (30.5%)	
Mixed	9 (1.6%)	15 (2.7%)	
Serous	25 (4.5%)	93 (16.8%)	
**Histologic grade, n (%)**			< 0.001
G1	80 (14.7%)	19 (3.5%)	
G2	85 (15.7%)	36 (6.6%)	
G3	109 (20.1%)	214 (39.4%)	
**Tumor invasion(%), n (%)**			0.027
< 50	154 (32.4%)	107 (22.5%)	
>= 50	105 (22.1%)	110 (23.1%)	
**Menopause status, n (%)**			0.209
Pre	21 (4.1%)	14 (2.8%)	
Peri	11 (2.2%)	6 (1.2%)	
Post	222 (43.8%)	233 (46%)	
**Diabetes, n (%)**			0.917
No	168 (37.1%)	161 (35.5%)	
Yes	64 (14.1%)	60 (13.2%)	

### Differential RNA sequence levels of TTK as a potential biomarker for differentiating endometrial cancer samples from normal samples

**Fig. 4:**
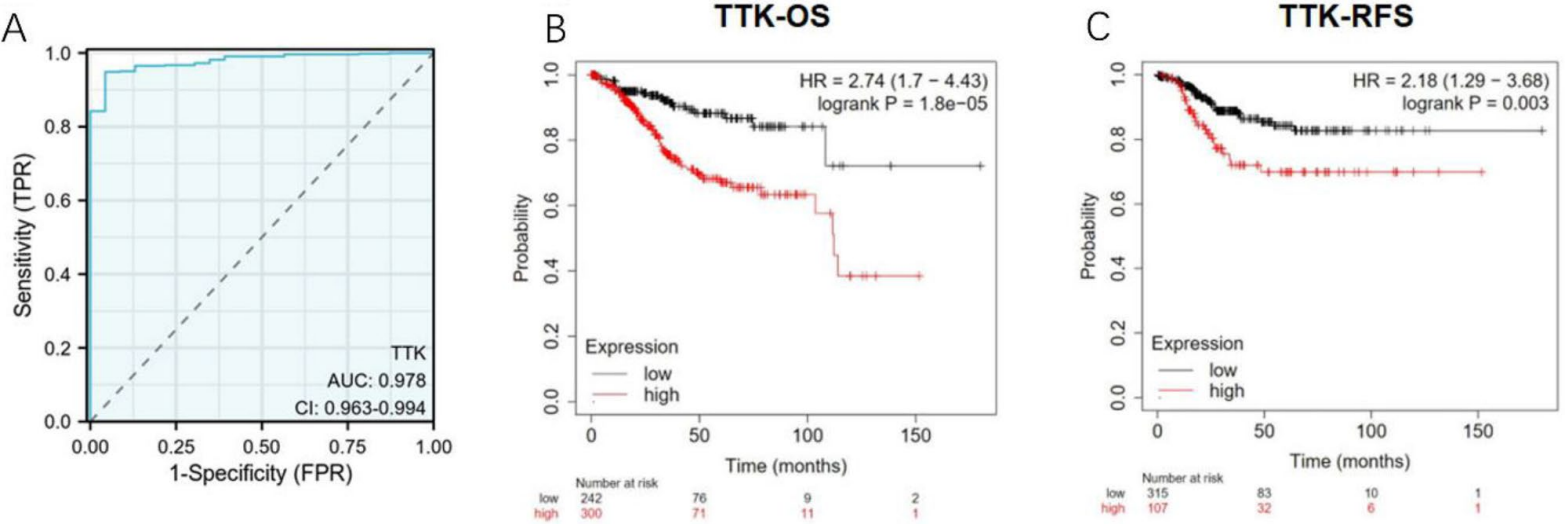
Diagnostic and prognostic value of TTK in endometrial cancer. **(A)** ROC curve analysis showing that TTK can distinguish endometrial cancer tissue from normal tissue with an AUC of 0.978; **(B)** K-M curves for OS in patients in the TCGA cohort stratified according to high vs. low TTK expression; **(C)** K-M curves for disease-free survival in patients in the TCGA cohort stratified according to high vs. low TTK expression

To evaluate the ability of the TTK to distinguish between endometrial cancer and normal tissue samples, ROC curve analysis was performed. As depicted in Fig. 4A, the ROC curve analysis yielded an impressive area under the curve (AUC) value of 0.978 (95% confidence interval: 0.963–0.994) for TTK. At the optimal cutoff of 1.4779, the TTK showed 94.9% sensitivity, 95.7% specificity, and 94.9% accuracy. Moreover, the positive predictive value was 99.8%, and the negative predictive value was 44%. Our results indicate that TTK holds substantial promise as a discerning biomarker for distinguishing endometrial cancer tissue from normal tissue.

### High TTK mRNA expression is associated with short OS and RFS

We examined the correlation between TTK mRNA expression and OS and recurrence-free survival (RFS) in endometrial cancer patients using Kaplan‒Meier analysis. As depicted in Fig. 4B, a compelling association was revealed where patients with high TTK expression had significantly shorter OS than did those with low TTK expression (36.63 vs. 108.37 months, P < 0.001). Furthermore, Fig. 4C demonstrated a significant decrease in RFS among patients with high TTK expression compared to those with low TTK expression (P = 0.003). These findings strongly indicate that high TTK mRNA expression is an adverse prognostic biomarker in endometrial cancer.

### PPI networks and functional annotations

**Fig. 5:**
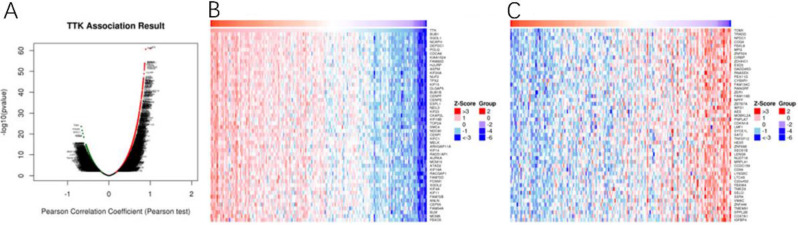
The TTK coexpressed genes in endometrial cancer were identified using the LinkedOmics database. **(A)** Pearson correlation analysis between TTK and differentially expressed genes in endometrial cancer; **(B)** heatmap showing the top 50 genes positively correlated with TTK; **(C)** heatmap showing the top 50 genes negatively correlated with TTK

**Fig. 6:**
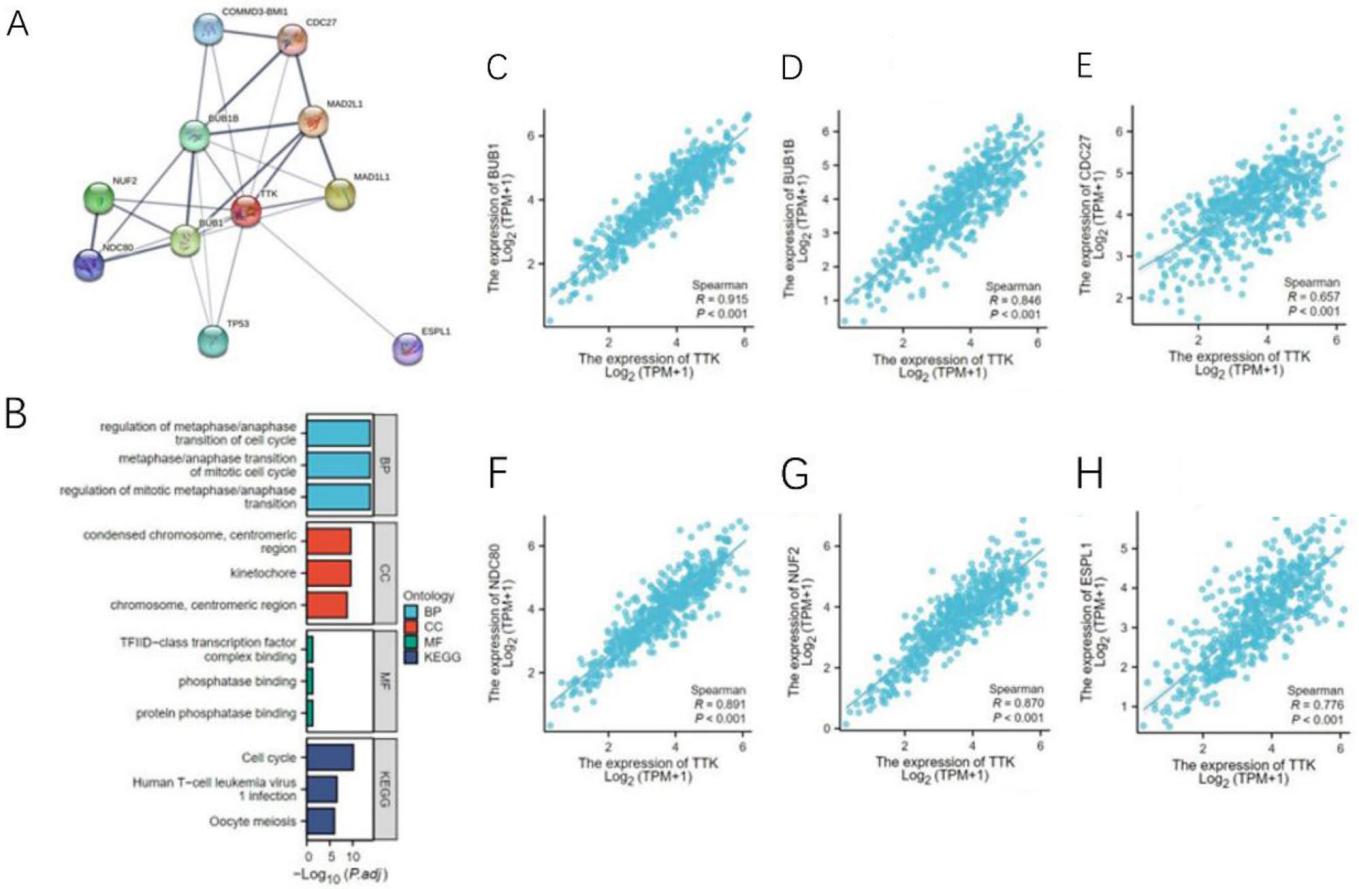
PPI network analysis and functional enrichment of TTK coexpressed genes. **(A)** PPI network of TTK and the top 10 coexpressed genes; the nodes represent proteins, and the edges represent interactions; **(B)** GO and KEGG pathway enrichment analyses showing that the 11 genes are involved in the cell cycle, mitosis, and mitophagy; **(C-H)** Pearson correlation analyses between TTK expression and the 10 hub genes in endometrial cancer

We analyzed TTK coexpression networks in EC using the LinkedOmics database (Fig. 5A). The database revealed that 10,401 genes exhibited significant correlations with TTK (P < 0.05). In EC, TTK was significantly negatively correlated with 6243 genes, while it was positively correlated with 4158 genes. Moreover, the heatmap illustrates the top 50 genes displaying noteworthy positive and negative correlations with TTK (Fig. 5B, C). To establish PPI networks and carry out functional annotations, we utilized the STRING database and conducted both GO and KEGG analyses. Figure 6A; Table 2 display the selection of the top ten functionally significant genes with substantial connectivity. These included *BUB1, BUB1B, CDC27, COMMD3-BMI1, ESPL1, MAD1L1, MAD2L1, NDC80, NUF2*, and *TP53*. Notably, alterations in TTK have been implicated in biological processes related to the regulation of mid-late cell cycle transition, mitotic regulation, and mid/late mitotic transition. The functional annotation further suggested the involvement of these genes in mitotic zone chromosomes, condensed chromosomes in the mitotic zone, and mitophagy (Fig. 6B). The correlation between TTK and the 10 hub genes was analyzed in the TCGA cohort (Fig. 6C-H).

**Table 2:** Detailed information on the TTK-related genes

Node	Annotation
BUB1	Mitotic checkpoint serine/threonine-protein kinase BUB1
BUB1B	Mitotic checkpoint serine/threonine-protein kinase BUB1 beta
CDC27	Cell division cycle protein 27 homolog
COMMD3-BMI1	annotation not available
ESPL1	Extra spindle pole bodies like 1
MAD1L1	Mitotic spindle assembly checkpoint protein MAD1
MAD2L1	Mitotic spindle assembly checkpoint protein MAD2A
NDC80	Kinetochore protein NDC80 homolog
NUF2	Kinetochore protein Nuf2
TP53	Cellular tumor antigen p53

### Correlation analysis of TTK expression and immune cell infiltration in endometrial cancer

**Fig. 7:**
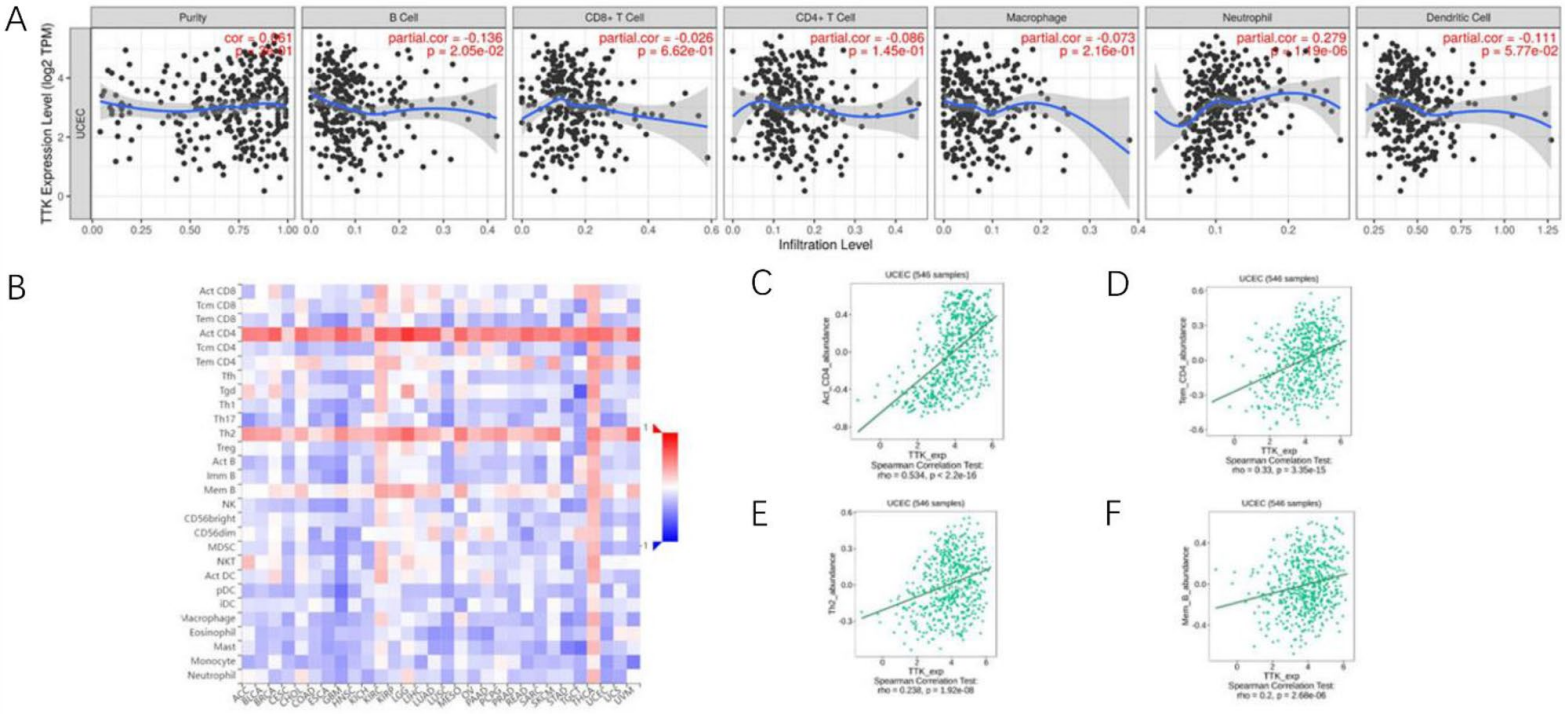
Correlations between TTK expression and immune infiltration level. **(A)** TTK expression is related to B cells and neutrophils in endometrial cancer; **(B)** relationships between the expression of TTK and 28 types of TILs across human cancers; **(C-F)** TTK was correlated with the abundance of CD4 + T cells, Tem CD4 + T cells, Th2 cells and Memory b cells

We analyzed the correlation between TTK expression and the infiltration of six tumor-infiltrating immune cells using the TIMER database. As shown in Fig. 7A, TTK expression was correlated with B cells (r=-0.136, P = 2.05e-02) and neutrophils (r = 0.279, P = 1.19e-06). Additionally, we assessed the relationship between TTK expression and 28 tumor-infiltrating lymphocytes (TILs) in the TISIB. Figure 7B shows the associations between TTK expression and the 28 TIL populations across various human cancers. Notably, as depicted in Fig. 7C-F, TTK expression exhibited significant correlations with the abundance of CD4 + T cells (r = 0.534, P < 2.2 × 10–16), Tem CD4 + T cells (r = 0.33, P = 3.35 × 10–15), Th2 cells (r = 0.238, P = 1.92 × 10 − 8), and memory B cells (r = 0.2, P = 2.68 × 10 − 6). These findings suggest that TTK may play a distinctive role in the immune infiltration of endometrial cancer.

### Correlation between TTK and immune cell infiltration

**Fig. 8:**
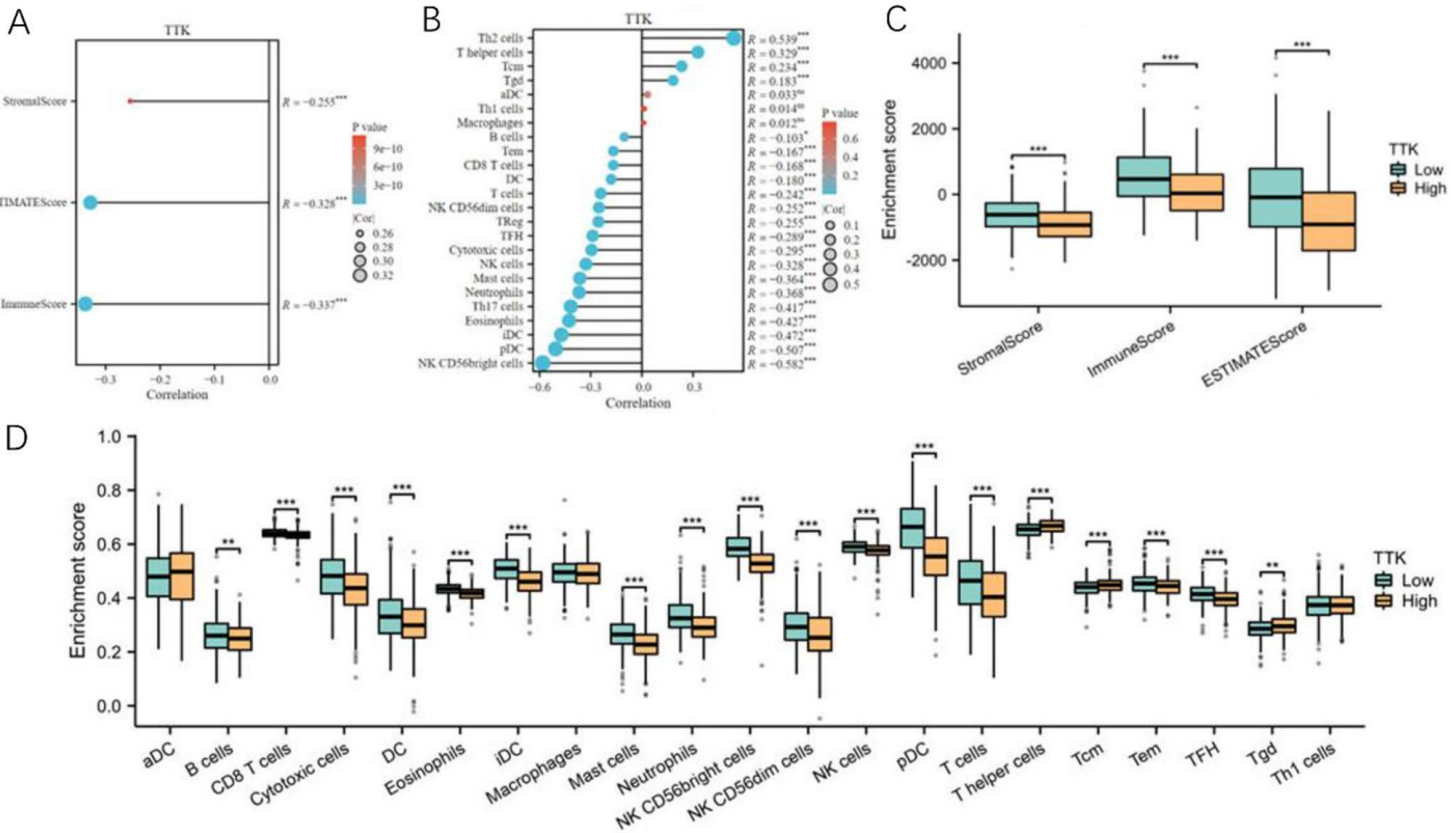
The relationship between TTK expression and immune cell infiltration. **(A)** the correlation between TTK expression and three immune-related scores (the immune score, ESTIMATEScore, and stromal score); **(B)** the associations between TTK expression and infiltration of various immune cell types are depicted; **(C)** enrichment scores of the three immune-related scores (immune score, ESTIMATEScore, and stromal score) in the TTK-high and TTK-low expression groups are displayed; **(D)** similarly, the enrichment scores of multiple infiltrating immune cell types in the TTK-high and TTK-low expression groups are shown; the statistical significance is indicated as ***P < 0.001, and “ns” represents “not significant”; the data are presented as the mean ± SD

In this study, we examined the relationship between TTK expression and immune cell infiltration using various analytical approaches. First, we employed the R Estimate package to calculate the ImmuneScore, ESTIMATEScore, and StromalScore, which are immune-related scores. The results demonstrated a negative correlation between TTK expression and the three immune-related scores (Fig. 8A). Next, we utilized the ssGSEA algorithm to investigate the association between TTK expression and infiltration of different immune cell types (Fig. 8B). Notably, we observed a positive correlation between TTK expression and the infiltration of Th2 cells and T helper cells. Conversely, TTK expression was negatively correlated with the percentage of CD56^bright^ NK cells. To further explore the impact of TTK expression on immune cell infiltration, we divided the TTK expression data into two groups. Subsequently, we calculated enrichment scores for immune cell infiltration in both the TTK-high and TTK-low expression clusters. Strikingly, the cluster with higher TTK expression exhibited significantly lower immune score, ESTIMATE score, and stromal score (Fig. 8C). Furthermore, enrichment scores for various cell types infiltrated by immune cells were negatively correlated with increased TTK expression (Fig. 8D). Taken together, these comprehensive analyses suggest a strong association between TTK expression and immune cell infiltration, highlighting the potential role of TTK in modulating the immune microenvironment in the context of the studied disease.

### Prognostic analysis based on TTK expression and immune cell infiltration in patients with EC

**Fig. 9:**
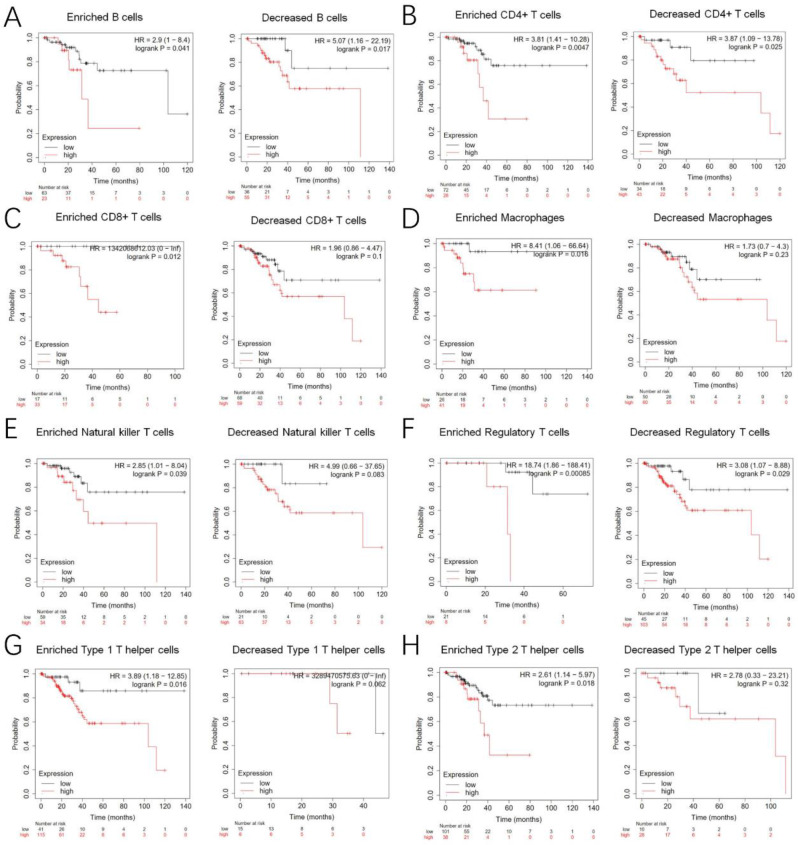
K-M survival curves comparing patients with high and low TTK expression in different immune cell subgroups within the EC cohort. (A-H)The correlations between TTK expression in these immune cell subgroups and overall survival (OS) in EC patients were estimated using CIBERSORT

Our study investigated the potential influence of TTK expression on the prognosis of EC patients, focusing on its association with immune infiltration and poor prognosis. We examined TTK expression levels in EC patients and stratified them based on relevant immune cell subsets using data from the Kaplan‒Meier plotter database. The analysis revealed that high TTK expression in EC patient cohorts, characterized by decreased B cells, CD4 + memory T cells, CD8 + memory T cells, and macrophages as well as increased NK T cells, was significantly correlated with poor prognosis (Fig. 9). Additionally, high TTK expression was associated with decreased Treg cells, Th1 cells, and Th2 cells. These findings suggest that elevated TTK expression may partially influence the prognosis of EC patients by affecting immune infiltration.

## Discussion

The prognosis of advanced EC is less optimistic than that of early endometrial cancer, and there is an urgent need to introduce new biomarkers for early detection [6]. Concurrently, the deployment of innovative methodologies is designed to enhance the sensitivity of chemotherapeutic agents, thereby substantially augmenting the prospects of treatment success for EC. This comprehensive strategy is expected to not only improve the cure rate but also have a positive impact on patient survival and quality of life.

In this study, we observed an upregulation of TTK mRNA expression across various tumors. The heightened expression of TTK in endometrial cancer was validated through multiple databases and cell lines. Clinicopathological characteristics and ROC curve analysis of TTK expression revealed positive correlations between TTK mRNA expression and tumor metastasis and between TTK mRNA expression and high TNM stage. This finding underscores the potential of TTK mRNA expression as a diagnostic biomarker for distinguishing endometrial cancer tissue from normal tissue. Additionally, Kaplan‒Meier analysis revealed that increased TTK mRNA expression was associated with a poorer prognosis, positioning TTK as a prospective biomarker for poor prognosis in patients with endometrial cancer. Our findings also suggest a unique role for TTK in immune infiltration in endometrial cancer.

TTK is a key spindle assembly checkpoint enzyme that regulates tumor cell proliferation across organisms. Aberrant TTK overexpression has been noted across cancer types, implicating its oncogenic role [26]. Numerous studies have demonstrated the carcinogenic effects of TTK in various human cancers, including triple-negative breast cancer, pancreatic cancer, and hepatocellular carcinoma [27–29]. Furthermore, TTK upregulation has been reported in different cancers and is associated with unfavorable prognosis [30]. However, the expression and prognostic value of TTK in endometrial cancer have not been extensively explored. In our study, using a pancancer analysis, we observed aberrant TTK mRNA expression in patients with various cancers, consistent with previous reports.

Recent studies and our results strongly indicate that TTK is a biomarker of poor prognosis across cancers [31]. In pancreatic ductal adenocarcinoma (PDAC), TTK knockdown substantially reduced proliferation and increased apoptosis in cell lines, highlighting its role in pancreatic cancer growth [28]. In non-small cell lung cancer (NSCLC), elevated TTK expression has been linked to an unfavorable prognosis [32]. Similarly, knockdown of TTK inhibited cell proliferation, migration, and tumorigenesis in NSCLC. Likewise, in other types of lung cancer, heightened TTK expression has been reported to promote cell migration and epithelial-mesenchymal transition (EMT), thereby enhancing metastatic potential and facilitating tumor metastasis [33, 34]. Additionally, TTK expression was elevated in hepatocellular carcinoma tissue compared to normal tissue, and functional studies confirmed TTK’s oncogenic activity. Notably, this intervention is associated with increased senescence and autophagy in tumor cells [35].

However, the prognostic value of TTK in endometrial cancer has not been explored. The positive correlation between TTK upregulation or metastasis and advanced TNM stage suggested that TTK is involved in endometrial cancer progression. Our Kaplan‒Meier and log-rank analyses revealed reduced survival in patients with high versus low TTK expression. Taken together, we conclude that TTK can indeed serve as a biomarker for predicting poor prognosis in patients with endometrial cancer.

Tumor development is closely linked to alterations in the tumor microenvironment (TME) [36]. Cancer cells can reprogram their microenvironment by releasing various factors that modify neighboring immune cells [37]. This interplay between cancer and immune cells creates a conducive environment that fosters tumor growth, promoting tumor survival and progression [38]. However, the relationship between TTKs and immune infiltration has not been explored. In this study, we observed a positive correlation between high TTK expression in EC and increased infiltration of helper T2 and helper T cells as well as a negative correlation with CD56^bright^ NK cells. Additionally, higher TTK expression was inversely correlated with the enrichment of various infiltrating immune cell types. Moreover, the influence of immune cell infiltration on TTK expression has a significant impact on EC patient survival. These findings suggest that TTK might serve as a promising target for future combination immunotherapy trials. Nonetheless, further research is warranted to elucidate the precise role of TTKs in the tumor immune microenvironment.

The role of TTK inhibitors has been extensively documented in numerous articles, and these inhibitors are currently in clinical use [12, 27, 29, 39–41]. The dual TTK/CLK2 inhibitor CC-671 provides a theoretical basis for overcoming ABCG2-mediated MDR in lung cancer patients [40]. In multiple myeloma, the TTK inhibitor OSU-13 can inhibit tumor growth in vitro and in vivo, improving patient prognosis [12]. In liver cancer, the TTK inhibitor AZ3146 can enhance DNA damage, increasing the sensitivity of cancer cells to radiation [41]. Additionally, the orally active small molecule inhibitor of TTK (CFI-402,257) effectively inhibits tumor growth and promotes the action of immune cells [29]. Some studies have also explored the impact of combining TTK inhibition with other treatment strategies, such as chemotherapy or targeted therapy. Theoretically, these combination approaches can enhance the efficacy of cancer treatment, potentially overcoming resistance mechanisms. In endometrial cancer, whether using immune checkpoint inhibitors alone or in combination with different drugs, significant breakthroughs can be achieved in cancer treatment, thereby extending the survival period of tumor patients [42, 43].

This study has several limitations that should be acknowledged. First, the investigation of TTK expression and its prognostic importance relied on an online public database; thus, it is crucial to validate these findings through further studies involving clinical samples. Second, in vivo or in vitro experiments are needed to fully elucidate the mechanistic role of TTK in immune infiltration. These experiments could enable further investigations of the specific underlying mechanisms involved.

## Conclusion

In summary, our study revealed that upregulated TTK mRNA and protein expression in endometrial cancer was positively correlated with metastasis and advanced TNM stage. These findings indicate TTK’s potential as a prognostic biomarker for identifying endometrial cancer patients with poor clinical outcomes. Our results also point to a possible role for TTK in immune infiltration.
